# Bull Semen Obtained on Beef Farms by Electroejaculation: Sperm Quality in the First Two Hours of Storing with Different Extenders and Holding Temperatures

**DOI:** 10.3390/ani13091561

**Published:** 2023-05-06

**Authors:** Santiago Pernas, Aitor Fernandez-Novo, Clara Barrajon-Masa, Patricia Mozas, Natividad Pérez-Villalobos, Bárbara Martín-Maldonado, Agustín Oliet, Susana Astiz, Sonia S. Pérez-Garnelo

**Affiliations:** 1 Department of Veterinary Medicine, School of Biomedical and Health Sciences, Universidad Europea de Madrid, 28670 Villaviciosa de Odon, Spain; 2 Centro de Selección y Reproducción Animal Colmenar Viejo (IMIDRA-CENSYRA), 28770 Colmenar Viejo, Spain; 3 Reproducción Animal (INIA-CSIC), 28040 Madrid, Spain

**Keywords:** BBSE, short–term storage, seminal parameters, CASA parameters, semen extenders

## Abstract

**Simple Summary:**

The contributions to the development and application of artificial insemination (AI) in beef cattle are essential to improve genetics and increasing farm productivity. Many factors affect sperm viability and fertilization capacity whether fresh, refrigerated or frozen–thawed semen doses are used. The aim of this study was to elucidate how different in vitro storage conditions affected semen quality in the first two hours after ejaculation. For this purpose, we studied three evaluation times from collection (<75, 75–105 and 105–120 min), two storage temperatures (refrigeration and room temperature), and two extenders (AndroMed^®^ and BIOXcell^®^) as well as the interaction of these factors on classical sperm parameters, CASA results and microbial growth on ejaculates collected under different farm conditions. We found that both extenders were suitable for seminal sample storage at both temperatures during these times. However, AndroMed^®^ induced a more curvilinear sperm movement, while BIOXcell^®^ stimulated straighter sperm motility, regardless of storage temperature.

**Abstract:**

Sperm quality decreases over time, so bull semen may need to be preserved after field collection. However, the effect of handling such semen samples from commercial farms and placing them in very short–term storage has not been elucidated. Therefore, ejaculate from 25 bulls from 1 dairy and 14 beef cattle farms were collected under farm conditions and evaluated for semen quality during the first two hours after collection. Two commercial extenders (AndroMed^®^ and BIOXcell^®^) and two different storage temperatures (5 °C and room temperature) were used to evaluate the influence on semen quality and sperm kinetics in ejaculates grouped into three evaluation times, based on time since collection (Time 1: <75 min, *n* = 7; Time 2: 75–105 min, *n* = 11; and Time 3: 105–120 min, *n* = 7). Classical semen parameters, sperm motion kinetics by CASA and colony-forming units were assessed. The differences between both extenders in curvilinear and straight–line velocities (VCL and VSL) for the different time groups (Time 2 and Time 3) were statistically significant for *p* < 0.05. AndroMed^®^ showed lower VSL, straightness and linearity in sperm compared to BIOXcell^®^ (*p* < 0.05). In conclusion, AndroMed^®^ induced more curvilinear movement, while BIOXcell^®^ stimulated straighter motility.

## 1. Introduction

On beef cattle farms, semen doses used in artificial insemination (AI) can be frozen–thawed, refrigerated or fresh [1,2,3], but regardless of conservation method, sperm preservation deals with different challenges that have a direct impact on fertilisation capacity, such as osmotic or pH changes, thermal shock and motility loss [4]. Some factors and circumstances from conservation processes that affect the fertilisation capacity of the semen doses [5] and sperm viability [6] have been described: sperm storage temperatures [7], length of long–term storage [7], freezing rates [8,9], sperm injury, [10], extenders used [1] and sperm motility [11,12]. In recent years, numerous efforts have been made to optimize semen preservation methods [13,14,15] and cryoprotectants [16] to preserve both membrane integrity [17] and sperm motility [18]. Some authors agree that storage time before final preservation of semen influences the viability of both refrigerated [19,20] and frozen–thawed semen [7,21] and has a negative impact on sperm progressive motility [22].

It is known that sperm motility decreases over time as the availability of nutrients in the medium decreases [23] and that the higher their metabolism the shorter their survival time, as has been shown in studies of medium- and long-term semen preservation [2], but there is scant information about the short-term storage (<2 h) of semen samples despite its relevance. Beyond studying semen quality under this time lapse (<2 h), it is crucial to determine whether the time from collection to evaluation or the conservation procedures could interfere with final seminal quality. It could have a negative impact on seminal quality and therefore on final fertility if used for AI. Moreover, this may be especially relevant for semen obtained in the field from commercial beef bulls, difficult-to-handle animals, endangered breeds, or wild bovine species [21]. Short-term storage may influence the final quality and reproductive efficiency of semen when used refrigerated or cryopreserved. In fact, studies of bull semen obtained at AI centres have pointed out these issues [24,25,26,27,28], thereby revealing that short-term cooling of bull semen doses after collection (instead of freeze-thawing) using an egg-yolk based extender [29,30] increased the conception rate. Consequently, further investigation into semen collection methods and handling to improve sperm longevity and fertilization ability was recommended [29,30,31]. On the other side, the demand for egg yolk replacement in extenders has increased in recent years due to the extremely wide variability of their composition based on their initial sources and the constant risk of contamination by bacteria or mycoplasma, which could be a source of endotoxins that could damage the fertilizing capacity of spermatozoa [32]. Therefore, our objective was to determine the effect of short-term storage, within 2 h after ejaculation, on the quality of bull semen samples obtained in commercial beef cattle farms by electroejaculation under field conditions and stored in vitro using different commercial soybean lecithin-based extenders and storage conditions. For this purpose, three different semen evaluation times from collection (<75, 75–105 and 105–120 min), two commercial extenders [AndroMed^®^ (Minitube, Tiefenbach, Germany) and BIOXcell^®^( IMV Technologies, L’Aigle, France)], and two storage temperatures (controlled ambient temperature and 5 °C) were compared. Semen quality was evaluated according to classical semen parameters, sperm motility and kinetics using Computer Assisted Sperm Analysis (CASA) and microbiological quality. These results form part of a research project focusing on different aspects of semen preservation, depending on the final aim. In previous studies [33,34] our group analyzed the impact of medium-term storage (2–24 h after collection) under different conditions on the semen quality in the frame of the bull breeding soundness evaluation (BBSE) and to classify correctly the bulls. In the current study, we analyzed the effects of the conditions on semen quality during the first two hours after collection because it may be crucial to decide if the semen is suitable for further preservation. These results will help to improve the handling conditions of bull semen samples in the very short-term after ejaculation under field conditions, which will be of great value when short-term storage is necessary for the cryopreservation of semen from high-value sires for transport to laboratories with the necessary freezing equipment.

## 2. Materials and Methods

### 2.1. Bulls and Herds

A total of 25 ejaculates, one per bull, from five different breeds (Limousine, Charolais, Blonde D’Aquitania, Spanish Black Iberian Avileña, Holstein Friesian) and crossbreeds were collected on 14 different commercial beef cattle farms and one dairy cattle farm located in central Spain. Bulls were free tested for infectious bovine rhinotracheitis, bovine viral diarrhoea, *Campylobacter foetus*, *Tritrichomonas foetus*, *Besnoitia besnoiti* and for tuberculosis, brucellosis, peripneumonia, and bovine leucosis (notifiable diseases). Bulls, aged between 24 and 108 months, were kept apart from dams for at least 15 days before electroejaculation. All of them, presented rectal a temperature <39.0 °C and scrotal circumference >34 cm. An ultrasound reproductive scanner revealed no pathologies (7.5 MHz transrectal transducer; SIUI CTS 800^®^; Shantou Institute of Ultrasonic Instruments Co., Ltd., Guangdong, China).

### 2.2. Ejaculate Collection and Sample Storage

Samples were obtained during a routine BBSE performed by the farm veterinarians following advised protocols [35] such that the study interventions were not considered by competent authorities to be experimental procedures but rather farming practises as expressed in the document PROEX 171/17 under the auspices of the Comunidad of Madrid (Jefa de la Sección Técnica I; Área de Protección Animal; Comunidad de Madrid, 21 November 2017; 5 years valid). Artificial vagina was rejected for semen collection due to required previous training, which is not possible for commercial farm bull sampling, usually once a year. Therefore, electro-ejaculation under respectful welfare conditions was performed, following our national guidelines [35]. In addition, electroejaculation is the method of choice proposed in Canadian, American and British guidelines for semen collection in the framework of a BBSE [36,37,38].

For sample collection, we followed the Spanish BBSE Guide [35]. Briefly, the preputial fur was cut, washed with a physiological saline solution and dried with sterile swabs. Feces were removed from the rectum and a massage on the accessory glands was performed before inserting the 75 mm transrectal probe (Electroejaculator Pulsator IV^®^, Lane Manufacturing, Denver, CO, USA). Electroejaculation was performed using the automatic program. Semen samples were collected in sterile 15 mL Falcon^®^ tubes immersed in a 50 mL tube with pre-warmed water (37 °C) to avoid thermal shocks. Each ejaculate was aliquoted (1:2 aliquots; 1 mL ejaculate and 2 mL extender) using two different extenders: AndroMed^®^ and BIOXcell^®^. Two aliquots (one per extender) were kept at a controlled ambient temperature (AT; 23–25 °C), and the other two at 5 °C until evaluation. Temperature was assessed by a data logger (176 H2; Testo, Barcelona, Spain). Samples were identified as AndroMed^®^ 5 °C (A5), AndroMed^®^ ambient temperature (AAT), BIOXcell^®^ 5 °C (B5) and BIOXcell^®^ ambient temperature (BAT). For the microbiological analysis, 400 µL from each aliquot were immediately frozen in liquid nitrogen. Depending on the arrival time of the samples at the laboratory after ejaculation for qualitative evaluation, the ejaculates were divided into three experimental groups: “Time 1” of assessment (<75 min after ejaculation; *n* = 7), “Time 2” (75–105 min; *n* = 11) and “Time 3” (105–120 min; *n* = 7).

### 2.3. Semen Quality Assessment

Semen assessment was performed at the referral laboratory (Centre of Selection and Animal Reproduction, IMIDRA–CENSYRA; Madrid, Spain) and microbiological analysis at Labocor SL (Madrid, Spain).

The parameters measured were sperm viability, normal sperm morphology, viable acrosome–intact spermatozoa, CASA kinetic parameters, pH and colony–forming units (CFU). Sperm viability (%) and normal sperm morphology (%) [36,37,38] were evaluated by eosin–nigrosine vital staining [39]. Live sperm percentages were obtained after counting 100 spermatozoa per slide in four slides per aliquot under bright–field microscopy (400×). The percentage of live acrosome–intact spermatozoa (%) was determined by a Giemsa overstaining procedure [39,40]. This triple stain technique highlights four categories of spermatozoa: live acrosome–intact (the key parameter assessed in our study), live acrosome–reacted or damaged, dead acrosome–intact, and dead acrosome–reacted or damaged. The percentages of each subpopulation were calculated by counting 100 spermatozoa per slide in two slides per aliquot under bright–field microscopy (1000×).

The CASA analyses were performed with a phase–contrast Nikon Eclipse Ci microscope, as described previously by our research group [33]. Briefly, images were transmitted to a computer for analysis using Sperm Class Analyzer software (SCA; Microptic Automatic Diagnostic Systems SL, Barcelona, Spain). Semen samples were diluted with the corresponding extender until a final sperm concentration of 6 million sperm/mL was reached, and 8 µL of diluted samples were deposited on a Spermtrack^®^ 20 µm chamber pre-warmed at 37 °C. At least, eight random fields were analysed such that a minimum of 2000 sperm was assessed per sample.

The software settings were those recommended by the manufacturer for analysis of bull sperm motility. The cell identification area was set at 28–70 µm^2^; sperm with a curvilinear velocity (VCL) <20 µm/s was considered immotile; 20–60 µm/s was considered slow; 60–110 µm/s was medium; and >110 µm/s was considered fast. Sperm with straightness (STR) >70 was considered to be progressively motile.

The percentages of sperm subpopulations evaluated according to kinetics are reflected in previous studies [33]. The kinetics of sp3 subpopulation (the fastest sperm group) is the only one described due to the main interest in fast spermatozoa [41]. The CASA kinetic parameters measured were total motility (%), progressive motility (%), VCL: curvilinear velocity (μm/s); VSL: straight line velocity (μm/s); VAP: average path velocity (μm/s); ALH: amplitude of lateral head displacement (μm); BCF: beat cross frequency (Hz); STR: straightness (VSL/VAP) × 100; LIN: linearity (VSL/VCL) × 100; and WOB: wobble (VAP/VCL) × 100.

The standard plate count per mL method was used to measure colony–forming units (CFUs) at Labocor S.L. (Madrid, Spain). Using the methodology described before [34]. Indicator pH paper strips (Whatman^®^ CS) were used (gradations of 0.2–0.3 in a pH range from 1.8 to 9.7).

### 2.4. Statistical Analysis

Data were analysed using SPSS^®^ v.25 (IBM, Armonk, NY, USA). Differences associated with *p* ≤ 0.05 were considered significant. Variables were assessed for normality with the Kolmogorov–Smirnov test. Analysis of variance (ANOVA) or the non–parametric Kruskal–Wallis test was used to assess the significance of differences among aliquots within the same time interval within a time group, creating four subsets of samples in each time-group (AndroMed^®^ 5 °C vs. AndroMed^®^ AT vs. BIOXcell^®^ 5 °C vs. BIOXcell^®^ AT), and among aliquots prepared with the same extender at a different time within an extender-group, for example: AndroMed^®^ 5 °C at Time 1, vs. Time 2 vs. Time 3).

A generalized linear mixed (GLM) model was used for the results of the variables: % sperm viability, % live and % live acrosome-intact sperm, % sperm morphology, % total motility, % progressive motility, microbiological quality in log_10_ CFU and pH value. In the GLM, the bull was included as a random factor and assessed for statistically significant effect. All interactions between the factors were taken into account: extender vs. storing temperature, extender vs. time, storing temperature vs. time, and extender vs. storing temperature vs. time. Inter- and intrasubject effect tests (Greenhouse–Geisser) were performed.

## 3. Results

At semen collection, the average age of the 25 ejaculated bulls was 41.7 ± 23.3 months, the scrotal circumference 40.3 ± 2.85 cm, and rectal temperature 37.8 ± 0.51 °C. The average ejaculate volume was 6.7 ± 3.83 mL, and collections were obtained at the first electroejaculation procedure in all bulls, except for four animals that needed two cycles. The actual temperature of the samples kept in refrigeration was 6.8 ± 1.48 °C (−0.4 ± 0.12 °C/min of cooling rate) and that of the samples kept at room temperature was 24.5 ± 1.77 °C.

### 3.1. Classical Semen Parameters

The results of the classical parameters (sperm viability, normal sperm morphology, live acrosome–intact spermatozoa and pH) are summarized in Table 1.

No statistical differences were found among the extender–temperatures combination within each assessed time group. Similarly, when comparing results among the three different time groups within each combination extender by temperature (for example AndroMed^®^ 5 °C Time 1, vs. at Time 2, vs. at Time 3), no statistical differences were found (*p* > 0.05).

### 3.2. CASA Kinetic Parameters

The results obtained for total sperm motility, progressive motility and percentage of sperm subpopulation type 3 (fast sperms) are summarized in Table 2.

These three parameters did not show statistical significance among time windows (1 vs. 2 vs. 3) within each extender–temperature combination (*p* > 0.05). At Time 3, the BIOXcell^®^ AT tended to show a higher percentage of fast spermatozoa compared to AndroMed^®^, achieving even 80% of fast spermatozoa.

The results obtained for curvilinear, straight line and average path sperm velocities (VCL, VSL and VAP, respectively) are summarized in Table 3.

Curvilinear velocity (VCL) values for AndroMed^®^, obtained at time group 2 (75–105 min after collection) were significantly higher than for BIOXcell^®^ at ambient temperature. The lowest VCL values were obtained from BIOXcell^®^ at 5 °C in the ejaculates evaluated at 105–120 min (Time 3). VSL values with BIOXcell^®^ at any temperature and time behaved better than with AndroMed^®^ although at Time 3 (105–120 min) the differences between extenders were only significant with AndroMed^®^ when the samples were stored at ambient temperature. Intra-extender analyses with BIOXcell^®^ AT revealed statistically significantly higher VSL values at Time 1 (<75 min) than at Time 3 (105–120 min; *p* = 0.041). The results observed for the amplitude of lateral head displacement (ALH) and beat cross frequency (BCF) are shown in Table 4.

For both parameters, the analyses performed showed that the samples extended with BIOXcell^®^ (regardless of storage temperature) showed lower values for ALH and higher values for BCF. These differences for BCF reached statistical significance at the earliest assessment (Time 1 or <75 min) with BIOXcell^®^ at room temperature and independently of the storage temperature with ejaculates that had been evaluated after 75 min (Time 2 and Time 3). These parameters did not differ significantly among time windows (1 vs. 2 vs. 3) within each extender – temperature combination (*p* > 0.05). The results of sperm straightness, linearity and wobble indexes are detailed in Table 5.

The three ratios were affected by the extender used at all time windows, with BIOXcell^®^ achieving higher values for STR, LIN and WOB. The intra-extender analysis with BIOXcell^®^ and AndroMed^®^ showed no statistical differences among the three analyzed time periods (*p* > 0.05).

### 3.3. Colony Forming Units

The microbiological quality results obtained for the colony-forming units (CFU) counts are summarized in Table 6. No statistical differences were observed with time, extender or temperature, or with interactions (*p* > 0.05).

## 4. Discussion

The extenders used in our study did not induce significant differences in the classic semen parameters: total and progressive motility and percentage of sperm subpopulation type 3. However, regarding other CASA kinetic parameters, we observed a more rectilinear movement pattern for samples extended with BIOXcell^®^ versus a more curvilinear and wavy movement for AndroMed^®^ samples regardless of time or storage temperature.

No significant differences were found between both holding temperatures (refrigeration and room temperature) with the two extenders used at any of the three evaluation times (Time 1: <75 min, Time 2: 75–105 min, and Time 3: 105–120 min). It has been reported that epididymal sperm from African buffalo is capable of surviving storage in a freezing media for at least 9 h at around 4 °C without any negative effect on progressive, total motility or acrosomal integrity [21]. Our results showed that this is true, during the three proposed storage periods, even when spermatozoa were exposed to glycerol at room temperature (controlled ambient temperature 23–25 °C), which is of great importance when transporting samples under field conditions to a laboratory for further processing.

According to previous studies [42,43,44,45,46], the chemical and physical properties of the extender may favor the progressive motility of the spermatozoa, so the physical characteristics of the extender could influence sperm function and, consequently, artificial insemination success [42]. Some authors [47,48] observed that extenders with an optimized soy–based concentration of 25% produced better sperm motility and viability (in bovine semen preserved at 5 °C at different time intervals). This issue could be definitive for actual fertility because an increased progressive motility relates to the fertilization capacity of spermatozoa [43,49]. In addition, breed variability could induce different motility values, as Hallap et al. [50] described for Holstein and Belgian Blue bulls, or Karthivaran and colleagues [43] for Jersey and Kangayam bulls. This could also influence differences found in the subpopulations of the ejaculate, as shown in the work of Víquez et al. [45]. 

Our study had certain limitations. The sperm came from different breeds, which may have triggered differences due to breed or individual variability. Unfortunately, we could not explain this variability because of the reduced size of the samples of ejaculates. It is also true that although sperm motility is commonly believed to be one of the most important characteristics for evaluating the potential fertility of ejaculated spermatozoa, it is not the only physiological function necessary for fertilizing an oocyte. Other functions such as membrane and acrosome integrity or ATP content are necessary for optimal fertility [51], issues that were not assessed in the current study.

The sperm subpopulations according to kinetics showed no statistically relevant differences but rather a tendency to increase the fraction of fast spermatozoa at Time 3 with BIOXcell^®^, which was previously shown by Víquez et al. [45] and Contri et al. [52]. As previously demonstrated [45], motility patterns with AndroMed^®^ showed a tendency toward a more curvilinear and wavy motion for sperm from different breeds of *Bos taurus* and *Bos indicus* collected using an artificial vagina at AI centres. Our results confirmed similar motility patters in semen obtained with an electroejaculator under field conditions. Viquez et al. [45] attributed these changes in sperm kinetics to the extender and Verstegen et al. [53] also found different responses in subpopulations when stimulated by different products [53]. Thus, breeds and extenders influenced the behaviour of the different subpopulations in the ejaculate.

AndroMed^®^ samples presented higher VCL values compared to BIOXcell^®^, at room temperature and in ejaculates evaluated 75–105 min after collection (Time 2). Thus, the movement of AndroMed^®^ was more curvilinear and wavier but less progressive or linear. These data are similar to those reported in other studies [45] with AndroMed^®^ showing higher VCL values and a better wave motion than Androstar^®^ or BTS. The high VCL and ALH values obtained with AndroMed^®^ suggested that the chemical composition or physical properties of the extender may have interfered with sperm motion from the time of addition. In this regard, some authors indicated that an increase in the viscosity of the extender due to the presence of larger molecules in its composition induces this type of curvilinear movement, which occurs naturally in cervical mucus [54]. The viscosity of cervical and oviductal mucus induced hyperactivation of spermatozoa [55,56], and the viscosity of the extender can similarly influence sperm motility [57,58]. This hyperactivity led to a change in motility, increasing VCL and ALH values and decreasing VSL [43,55]. This hyperactivation can be positive because it favours the penetration of the sperm into the oocyte [59], but if this activation occurs early, it can be unstable [60] and undesirable because it would reduce the spermatozoa lifespan and fertilisation capacity [53].

On the other hand, BIOXcell^®^ velocity outputs showed a more rectilinear motion with higher VSL values. In their studies with BIOXcell^®^, Chaudhari et al. [61] also showed more straightness and linearity in the movement of spermatozoa [61] as did the work of Celeghini et al. [54] and Vera-Munoz et al. [62], where BIOXcell^®^ also showed changes in motility. This may have been due to the different density or viscosity of the extender and the presence of smaller particles [54], which induced more straight movements [63] because the spermatozoa tail was less curved and remained straighter [64,65]. The viscosity of the fluid influenced the type of movement of the spermatozoa [56] as it did the type of extender [54,63,66]. However, the correlation between rectilinear or curvilinear movement with improved fertility was not clear. Amann and Waberski [67] suggested that sperm movement assessment cannot be related to fertilising ability, while others positively correlate motility parameters (VSL, VCL, VAP) with sperm fertility [68,69,70].

However, sperm kinetics using CASA are usually examined after diluting raw semen in a complex extender, giving it a composition different from the viscosity or chemical composition of sperm fluid they would be exposed to in a female. Extender conditions affect sperm motion and function; therefore, in vitro measurements of sperm motion can reflect capability within highly variable environment in a female’s reproductive tract [67].

Similarly, the values for STR, LIN, WOB and BFC were higher for BIOXcell^®^ (regardless of temperature and storage time). We could suggest that BIOXcell^®^ preserves a more linear sperm trajectory and straighter movement with less lateral displacement of the head (ALH). The thesis by Gallardo [71] showed a correlation between these high STR and low ALH values with linear trajectories. This type of spermatozoa movement between the walls of the oviduct favours a more progressive movement [44] because the ribbed anatomy of the oviduct walls limits circular but favours progressive and rectilinear movement [55,72,73].

ALH measures the vigour of flagellar beating in conjunction with the frequency of cell rotation [53], which has been associated with the ability of sperm to penetrate cervical mucus and fuse with oocytes. ALH values were higher for AndroMed^®^, both at 5 °C and at room temperature and in the three time groups. Considering that the higher the ALH, the lower the LIN, STR and WOB variables [74], it could be said that AndroMed^®^ induces more curvilinear spermatozoa movement. High ALH values and non–linear trajectories are similar to a pre–capacitated or hyperactivated movement pattern [43,75]. Peña et al. [70] pointed out that fast and linearly moving spermatozoa are more likely to reach and fertilize the oocyte [49,70]. On the other hand, only spermatozoa with good, progressive motility and a high amplitude of lateral head displacement are able to penetrate oestrous cervical mucus [76,77,78] and some studies have shown a positive correlation among total number of motile sperm, VCL and fecundity [53]. VCL alone may also predict fertility [79] and bulls characterized with higher field fertility displayed lower VSL, STR and LIN [80]. Moreover, it has been shown that ALH is higher in high-fertility bulls compared to low fertility bulls, and consistent with this observation LIN, STR, WOB and VSL were lower in high fertility versus low-fertility sires [81]. However, the effect on the fertility of enhanced straight sperm movement (obtained with BIOXcell^®^) or enhanced curvilinear sperm velocity (AndroMed^®^) is controversial, and further studies are required to clarify this issue.

Finally, colony forming units did not show any statistical difference regardless of time, extender or temperature. However, bacterial growth was lower at Time 3 (105–120 min). The activity of antibiotics depends on many factors, including the time it takes for them to exert their antimicrobial action [82]. Moreover, soy lecithin–based extenders (AndroMed^®^, BIOXcell^®^) present a lower microbiological risk [63] and are more hygienic because they are free of animal components (such as egg yolk), which are more conducive to bacterial growth [1] infections, and the production of toxins [83]. Thus, they provide better sanitary conditions [32]. Therefore, it appears that the microbiological quality of the semen ejaculates obtained in the field can be controlled for at least 2 h after ejaculation.

In summary, if we consider that faster spermatozoa with a more linear movement can have higher fertilization capacity, BIOXcell^®^ could be the extender of choice for cattle. Meanwhile, according to other authors, AndroMed^®^ would be the more suitable extender for fast spermatozoa with less linear but more curvilinear movement. Therefore, further studies are needed.

## 5. Conclusions

The commercial extenders (AndroMed^®^ and BIOXcell^®^) subjected to two storage temperatures (5 °C vs. room temperature) did not reveal significant differences during the first 2 h after sampling in classical parameters. However, a different behaviour in CASA kinetic parameters was detected, but further research is required to clarify the relevance of this. In any case, under these conditions, semen quality was satisfactorily preserved, demonstrating that semen quality was adequate after using the extenders under experimental conditions.

## Figures and Tables

**Table 1:** Bull sperm viability, normal sperm morphology, live acrosome–intact spermatozoa and pH for the three experimental groups (according to time of assessment) kept with two commercial extenders at two storage temperatures.

		Sperm Viability (%)		Normal Sperm Morphology (%)		Live Acrosome-Intact Spermatozoa (%)		pH	
		Average± SD	*p* Value	Average ± SD	*p* Value	Average ± SD	*p* Value	Average ± SD	*p* Value
Time 1 (<75 min) *n* = 7	AndroMed^®^ 5 °C	66.2 ± 16.06	0.63	87.8 ± 4.70	0.87	61.0 ± 11.60	0.70	6.5 ± 0.31	0.51
	AndroMed^®^ AT	70.6 ± 17.60		86.4 ± 8.73		56.0 ± 21.63		6.4 ± 0.39	
	BIOXcell^®^ 5 °C	66.7 ± 11.47		86.3 ± 7.34		51.4 ± 25.15		6.7 ± 0.29	
	BIOXcell^®^ AT	65.6 ± 16.69		85.0 ± 6.66		47.6 ± 21.17		6.7 ± 0.27	
Time 2 (75–105 min) *n* = 11	AndroMed^®^ 5 °C	65.9 ± 13.40	0.88	80.8 ± 10.39	0.78	58.4 ± 11.07	0.38	6.3 ± 0.25	0.84
	AndroMed^®^ AT	67.7 ± 10.37		80.6 ± 15.31		60.0 ± 9.10		6.4 ± 0.33	
	BIOXcell^®^ 5 °C	62.4 ± 14.78		79.1 ± 15.19		54.9 ± 12.07		6.5 ± 0.37	
	BIOXcell^®^ AT	67.4 ±10.81		79.7 ± 13.75		51.6 ± 14.63		6.4 ± 0.32	
Time 3 (105–120 min) *n* = 7	AndroMed^®^ 5 °C	73.0 ± 7.01	0.20	81.9 ± 7.59	0.93	52.9 ± 15.90	0.20	6.5 ± 0.31	0.99
	AndroMed^®^ AT	77.8 ± 6.08		82. ± 8.07		68.9 ± 12.94		6.5 ± 0.28	
	BIOXcell^®^ 5 °C	70.2 ± 7.85		82.6 ± 7.72		55.1 ± 16.36		6.4 ± 0.39	
	BIOXcell^®^ AT	71.6 ± 5.20		80.9 ± 6.21		58.1 ± 14.30		6.4 ± 0.37	

Abbreviations: SD = standard deviation; AT = ambient temperature.

**Table 2:** Bull total sperm motility (percentage), progressive motility (percentage) and sperm subpopulation type 3 (percentage), measured by CASA methodology, for the three experimental groups (according to the time of assessment) kept with two commercial extenders at two storage temperatures.

		Total Motility (%)		Progressive Motility (%)		Sperm Subpopulation Type 3 (Fast, %)	
		Average ± SD	*p* Value	Average ± SD	*p* Value	Average ± SD	*p* Value
Time 1 (<75 min) *n* = 7	AndroMed^®^ 5 °C	77.6 ± 9.59	0.38	71.6 ± 8.99	0.06	75.9 ± 9.42	0.34
	AndroMed^®^ AT	70.4 ± 16.10		57.4 ± 18.77		62.6 ± 26.31	
	BIOXcell^®^ 5 °C	81.3 ± 12.71		77.4 ± 12.02		77.7 ± 14.17	
	BIOXcell^®^ AT	78.2 ± 10.47		74.5 ± 11.94		75.7 ± 12.53	
Time 2 (75–105 min) *n* = 11	AndroMed^®^ 5 °C	69.7 ± 20.15	0.83	62.5 ± 20.12	0.88	66.3 ± 19.49	0.96
	AndroMed^®^ AT	76.7 ± 14.93		65.0 ± 17.61		70.9 ± 15.43	
	BIOXcell^®^ 5 °C	73.2 ± 19.14		68.4 ± 19.79		70.2 ± 19.30	
	BIOXcell^®^ AT	71.2 ± 19.73		65.6 ± 19.09		67.9 ± 20.57	
Time 3 (105–120 min) *n* = 7	AndroMed^®^ 5 °C	78.9 ± 7.90	0.27	71.8 ± 10.98	0.43	73.9 ± 9.15	0.10
	AndroMed^®^ AT	81.4 ± 5.35		71.6 ± 8.38		73.8 ± 8.52	
	BIOXcell^®^ 5 °C	81.8 ± 5.03		76.6 ± 7.06		79.2 ± 8.89	
	BIOXcell^®^ AT	85.1 ± 3.66		79.1 ± 5.98		81.9 ± 3.19	

Abbreviations: SD = standard deviation; AT = ambient temperature.

**Table 3:** Bull sperm curvilinear velocity (VCL, μm/s), straight line velocity (VSL, μm/s) and average path velocity (VAP, μm/s) measured by CASA methodology, for the three experimental groups (according to time of assessment) kept with two commercial extenders at two storing temperatures.

		VCL		VSL		VAP	
		Average ± SD	*p* Value	Average ± SD	*p* Value	Average ± SD	*p* Value
Time 1 (<75 min) *n* = 7	AndroMed^®^ 5 °C	215.6 ± 26.56	0.21	71.0 ± 14.27 ^a^	**<0.001**	114.6 ± 6.72	0.09
	AndroMed^®^ AT	209.5 ± 35.00		64.2 ± 12.57 ^a^		111.0 ± 12.33	
	BIOXcell^®^ 5 °C	194.8 ± 14.52		105.5 ± 7.35 ^b^		121.0 ± 6.27	
	BIOXcell^®^ AT	196.2 ± 11.61		105.0 ± 5.18 ^b^		120.6 ± 4.14	
Time 2 (75–105 min) *n* = 11	AndroMed^®^ 5 °C	212.5 ± 11.67 ^ab^	**<0.001**	75.6 ± 10.13 ^a^	**<0.001**	114.2 ± 5.59	0.22
	AndroMed^®^ AT	225.9 ± 14.65 ^b^		60.7 ± 6.82 ^a^		118.6 ± 8.38	
	BIOXcell^®^ 5 °C	194.1 ± 17.72 ^a^		103.3 ± 7.09 ^b^		120.4 ± 7.55	
	BIOXcell^®^ AT	197.3 ± 16.34 ^a^		100.5 ± 9.04 ^b^		119.2 ± 7.90	
Time 3 (105–120 min) *n* = 7	AndroMed^®^ 5 °C	194.6 ± 27.52 ^ab^	**0.05**	76.5 ± 8.50 ^ab^	**<0.001**	104.0 ± 12.99	0.16
	AndroMed^®^ AT	205.0 ± 33.25 ^a^		63.4 ± 7.13 ^a^		107.1 ± 14.89	
	BIOXcell^®^ 5 °C	180.4 ± 22.81 ^b^		93.4 ± 14.37 ^b^		109.2 ± 14.57	
	BIOXcell^®^ AT	200.9 ± 15.51 ^ab^		94.2 ± 6.79 ^b^		114.8 ± 4.58	

Abbreviations: SD = standard deviation; AT = ambient temperature; VCL = curvilinear velocity, μm/s; VSL = straight line velocity, μm/s; VAP = average path velocity, μm/s. Superscripts (a, b) meant statistical significance (*p* < 0.05) between groups by ANOVA test.

**Table 4:** Bull sperm lateral head displacement (ALH, μm) and beat cross frequency (BCF, Hz) measured by CASA methodology for the three experimental groups (according to time of assessment) kept with two commercial extenders at two storage temperatures.

		ALH		BCF	
		Average ± SD	*p* Value	Average ± SD	*p* Value
Time 1 (<75 min) *n* = 7	AndroMed^®^ 5 °C	5.4 ± 1.05	**0.03**	17.4 ± 4.43 ^ac^	**<0.001**
	AndroMed^®^ AT	5.4 ± 1.14		16.4 ± 3.84 ^a^	
	BIOXcell^®^ 5 °C	4.2 ± 0.66		25.1 ± 3.07 ^bc^	
	BIOXcell^®^ AT	4.1 ± 0.51		26.4 ± 2.47 ^b^	
Time 2 (75–105 min) *n* = 11	AndroMed^®^ 5 °C	5.3 ± 0.55 ^a^	**<0.001**	18.6 ±3.37 ^a^	**<0.001**
	AndroMed^®^ AT	5.8 ± 0.44 ^a^		16.9 ± 2.60 ^a^	
	BIOXcell^®^ 5 °C	4.2 ± 0.78 ^b^		25.0 ± 3.08 ^b^	
	BIOXcell^®^ AT	4.2 ± 0.79 ^b^		26.1 ± 3.21 ^b^	
Time 3 (105–120 min) *n* = 7	AndroMed^®^ 5 °C	4.8 ± 0.59 ^ab^	**0.02**	18.2 ± 2.88 ^ab^	**<0.001**
	AndroMed^®^ AT	5.2 ± 0.82 ^a^		17.2 ± 2.68 ^a^	
	BIOXcell^®^ 5 °C	4.1 ± 0.47 ^b^		22.4 ± 3.99 ^ab^	
	BIOXcell^®^ AT	4.6 ± 0.58 ^ab^		23.5 ± 2.57 ^b^	

Abbreviations: SD = standard deviation; AT = ambient temperature; ALH = amplitude of lateral head displacement, μm; BCF = beat cross frequency, Hz. Superscripts (a, b, c) meant statistical significance (*p* < 0.05) between groups by ANOVA test.

**Table 5:** Bull sperm straightness (STR), linearity (LIN) and wobble (WOB) indexes measured by CASA methodology, for the three experimental groups (according to time of assessment) kept with two commercial extenders at two storing temperatures.

		STR		LIN		WOB	
		Average ± SD	*p* Value	Average ± SD	*p* Value	Average ± SD	*p* Value
Time 1 (<75 min) *n* = 7	AndroMed^®^ 5 °C	62.9 ± 14.03 ^a^	**<0.001**	35.3 ± 11.23 ^ab^	**<0.001**	54.3 ± 5.31 ^a^	**<0.001**
	AndroMed^®^ AT	60.1 ± 15.54 ^a^		33.6 ± 11.74 ^a^		54.1 ± 4.43 ^a^	
	BIOXcell^®^ 5 °C	86.8 ± 3.37 ^b^		55.4 ± 5.15 ^b^		63.2 ± 4.35 ^bc^	
	BIOXcell^®^ AT	86.9 ± 4.51 ^b^		55.0 ± 4.47 ^b^		62.6 ± 3.52 ^ac^	
Time 2 (75–105 min) *n* = 11	AndroMed^®^ 5 °C	67.0 ± 7.61 ^a^	**<0.001**	37.5 ± 6.33 ^a^	**<0.001**	54.6 ± 3.36 ^a^	**<0.001**
	AndroMed^®^ AT	53.5 ± 7.06 ^a^		29.1 ±4.26 ^a^		53.2 ± 2.24 ^a^	
	BIOXcell^®^ 5 °C	85.5 ± 3.35 ^b^		54.6 ± 5.44 ^b^		63.3 ± 5.91 ^b^	
	BIOXcell^®^ AT	84.3 ± 3.27 ^b^		52.9 ± 6.80 ^b^		62.0 ± 6.49 ^b^	
Time 3 (105–120 min) *n* = 7	AndroMed^®^ 5 °C	74.1 ± 4.27 ^ab^	**<0.001**	41.2 ± 3.70 ^ab^	**<0.001**	54.5 ± 2.58 ^a^	**<0.001**
	AndroMed^®^ AT	61.8 ± 10.56 ^a^		33.5 ± 6.91 ^a^		53.2 ± 2.83 ^a^	
	BIOXcell^®^ 5 °C	84.9 ± 2.63 ^b^		52.8 ± 4.52 ^b^		61.5 ± 3.81 ^b^	
	BIOXcell^®^ AT	82.3 ± 5.91 ^b^		48.9 ± 5.69 ^b^		58.4 ± 3.43 ^ab^	

Abbreviations: SD = standard deviation; AT = ambient temperature; STR = straightness (VSL/VAP) × 100; LIN = linearity (VSL/VCL) × 100; and WOB = wobble (VAP/VCL) × 100. Superscripts (a, b, c) mean statistical significance (*p* < 0.05) between groups by an ANOVA test.

**Table 6:** Colony-forming units (CFU) for the three experimental groups (according to time of assessment) kept with two commercial extenders at two storing temperatures.

		CFU	
		Average ± SD	*p* Value
Time 1 (<75 min) *n* = 7	AndroMed^®^ 5 °C	35,991.4 ± 66,972.79	0.93
	AndroMed^®^ AT	26,034.3 ± 34,069.42	
	BIOXcell^®^ 5 °C	59,985.7 ± 96,667.35	
	BIOXcell^®^ AT	20,751.4 ± 34,282.38	
Time 2 (75–105 min) *n* = 11	AndroMed^®^ 5 °C	38,845.5 ± 80,382.39	0.59
	AndroMed^®^ AT	25,898.2 ± 65,293.44	
	BIOXcell^®^ 5 °C	289,349.1 ± 899,746.41	
	BIOXcell^®^ AT	260,472.7 ± 842,348.47	
Time 3 (105–120 min) *n* = 7	AndroMed^®^ 5 °C	24,411.4 ± 59,823.48	0.98
	AndroMed^®^ AT	4422.9 ± 7075.88	
	BIOXcell^®^ 5 °C	4791.4 ± 7334.11	
	BIOXcell^®^ AT	4414.3 ± 9534.72	

Abbreviations: SD = standard deviation; AT = ambient temperature; CFU = colony forming units.

## Data Availability

Data are contained within the article, and raw data are available on request by the authors.
